# Pharmacophore-Based Virtual Screening, Quantum Mechanics Calculations, and Molecular Dynamics Simulation Approaches Identified Potential Natural Antiviral Drug Candidates against MERS-CoV S1-NTD

**DOI:** 10.3390/molecules26164961

**Published:** 2021-08-17

**Authors:** Thamer A. Bouback, Sushil Pokhrel, Abdulaziz Albeshri, Amal Mohammed Aljohani, Abdus Samad, Rahat Alam, Md Saddam Hossen, Khalid Al-Ghamdi, Md. Enamul Kabir Talukder, Foysal Ahammad, Ishtiaq Qadri, Jesus Simal-Gandara

**Affiliations:** 1 Department of Biological Science, Faculty of Science, King Abdul-Aziz University, Jeddah 21589, Saudi Arabia; tbouback@kau.edu.sa (T.A.B.); aalbishri0061@stu.kau.edu.sa (A.A.); amal.m.aljohany@gmail.com (A.M.A.); kalghamdy@kau.edu.sa (K.A.-G.); 2 Department of Biomedical Engineering, State University of New York (SUNY), Binghamton, NY 13902, USA; sushilpokhrel@binghamton.edu; 3 Department of Genetic Engineering and Biotechnology, Faculty of Biological Science and Technology, Jashore University of Science and Technology, Jashore 7408, Bangladesh; kazisamad50@gmail.com (A.S.); rahatalam1643@gmail.com (R.A.); hossensaddam621@gmail.com (M.S.H.); talukder.mek@gmail.com (M.E.K.T.); 4 Laboratory of Computational Biology, Biological Solution Centre (BioSol Centre), Jashore 7408, Bangladesh; 5 Department of Microbiology, Faculty of Life Sciences and Medicine, Zhejiang Sci-Tech University, Hangzhou 310018, China; 6 Nutrition and Bromatology Group, Department of Analytical Chemistry and Food Science, Faculty of Food Science and Technology, University of Vigo—Ourense Campus, E-32004 Ourense, Spain

**Keywords:** MERS-CoV, S1-NTD, pharmacophore modeling, ADME, quantum mechanical calculation, DFT, FMO, MM/GBSA, molecular docking, Molecular Dynamics Simulation, HOMO, LUMO

## Abstract

Middle East respiratory syndrome coronavirus (MERS-CoV) is a highly infectious zoonotic virus first reported into the human population in September 2012 on the Arabian Peninsula. The virus causes severe and often lethal respiratory illness in humans with an unusually high fatality rate. The N-terminal domain (NTD) of receptor-binding S1 subunit of coronavirus spike (S) proteins can recognize a variety of host protein and mediates entry into human host cells. Blocking the entry by targeting the S1-NTD of the virus can facilitate the development of effective antiviral drug candidates against the pathogen. Therefore, the study has been designed to identify effective antiviral drug candidates against the MERS-CoV by targeting S1-NTD. Initially, a structure-based pharmacophore model (SBPM) to the active site (AS) cavity of the S1-NTD has been generated, followed by pharmacophore-based virtual screening of 11,295 natural compounds. Hits generated through the pharmacophore-based virtual screening have re-ranked by molecular docking and further evaluated through the ADMET properties. The compounds with the best ADME and toxicity properties have been retrieved, and a quantum mechanical (QM) based density-functional theory (DFT) has been performed to optimize the geometry of the selected compounds. Three optimized natural compounds, namely Taiwanhomoflavone B (Amb23604132), 2,3-Dihydrohinokiflavone (Amb23604659), and Sophoricoside (Amb1153724), have exhibited substantial docking energy >−9.00 kcal/mol, where analysis of frontier molecular orbital (FMO) theory found the low chemical reactivity correspondence to the bioactivity of the compounds. Molecular dynamics (MD) simulation confirmed the stability of the selected natural compound to the binding site of the protein. Additionally, molecular mechanics generalized born surface area (MM/GBSA) predicted the good value of binding free energies (ΔG bind) of the compounds to the desired protein. Convincingly, all the results support the potentiality of the selected compounds as natural antiviral candidates against the MERS-CoV S1-NTD.

## 1. Introduction

Coronavirus (CoVs) is a member of the family Coronaviridae, containing a single-stranded positive-sense RNA genome [+ssRNA] that has a length between ~27 kb to 32 kb. The virus causes illness ranging from the common cold to more severe diseases in humans and animals [1]. Genetically diverse coronaviruses cycles in nature among its three principal hosts, which are the natural host (bats, mice), intermediate host (camels, masked palm civets, swine, dogs, and cats), and humans [2]. Four common human coronaviruses (HCoVs) NL63 and 229E (α-CoVs); OC43 and HKU1 (β-CoVs) circulate widely in the human population, each capable of causing severe disease ranging from common colds to self-limiting upper respiratory infections in immunocompetent people.

Severe acute respiratory syndrome coronavirus (SARS-CoV), SARS-CoV-2, and MERS-CoV are other known human beta CoVs (β-CoVs) capable of causing epidemics [3,4]. They are zoonotic in characters and infections with the virus resulting in various clinical severity featuring respiratory and extra-respiratory manifestations [5]. The number of global people infected with coronavirus has risen rapidly, which began with the pandemic of SARS-CoV in 2003, followed by the MERS-CoV in 2012 and, most recently, the SARS-CoV-2 outbreaks with a fatality rate of ~10%, ~35%, and 0.1% to over 25%, respectively [6,7]. Among the animal coronaviruses, MERS-CoV has the highest fatality rate in humans and animals, but effective antiviral candidates are not available to treat the infection caused by the pathogen.

Receptor recognition is an initial and key step of a virus to infections into the host cells. MERS-CoV class I membrane fusion trimeric S glycoprotein of the virion can recognize host receptor that mediates entry into the cells. The S trimer of highly pathogenic MERS-CoV can recognize the host cellular receptor dipeptidyl peptidase 4 (DPP4), resulting in membrane fusion and viral entry [8]. The trimeric ectodomain segment of MERS-CoV-S protein can be divided into two subunits, the first one is the receptor binding S1 subunit and another one is the membrane-fusion S2 subunit. The receptor binding S1 subunit of the virus can also be divided into two independent domains, namely the N-terminal domain (S1-NTD) and C-terminal domain (S1-CTD), which can function as a receptor-binding domain (RBD) for the S protein [9]. The S1-NTD of MERS-CoV can identify specific sugar moieties upon primary attachment and help in the prefusion-to-post fusion transition, which is critical in determining tissue tropism, host ranges, and cross-species infection [10]. Therefore, targeting the S1-NTD of the MERS-CoV S protein can inhibit the primary attachment to the host and block the prefusion-to-post fusion transition and will be an effective prophylactic against the virus [11].

The S1-NTD targeting natural compounds with potent inhibitory activity can be a focus on the improvement of therapeutic interventions of the virus [12]. Many studies reported different neutralizing antibodies and chemically synthesized compounds as drug candidates previously, for example, folic acid showed activity against NTD from the mammalian expression medium [10,13]. Sometimes, this type of antibody can induce resistance against the virus and chemically synthesized compounds can causes adverse side effect of the host [14]. Natural compounds having low toxicity and side effect can be developed as antiviral candidates by targeting S1-NTD that will be novel therapeutics for MERS-CoV [15]. We thus sought to identify potential natural antiviral drug candidates against the MERS-CoV by targeting S1-NTD.

Nowadays, computer-aided drug design (CADD) has become an effective and powerful technique in different therapeutic development. The technique has helped to overcome the long-term and expensive process that costs billions of dollars previously during drug design and development [16]. The importance of the in-silico drug design technique is greater than ever before in the modern drug design process [17]. Therefore, the study utilized different in-silico technique includes pharmacophore modeling, virtual screening, molecular docking, ADMET, QM calculation, MD simulation, and MM/GBSA to identify effective and potential natural drug candidates against MERS-CoV.

## 2. Results

### 2.1. Results of Pharmacophore Modeling

Pharmacophore can be defined as an ensemble of common steric and electronic chemical features that indicates a compound-specific mode of action to the active site of a targeted biological macromolecule. The pharmacophore features can be observed during ligand–protein interaction and helps in screening a large chemical database for retrieving novel scaffolds as a lead compound [18].

**Figure 1:**
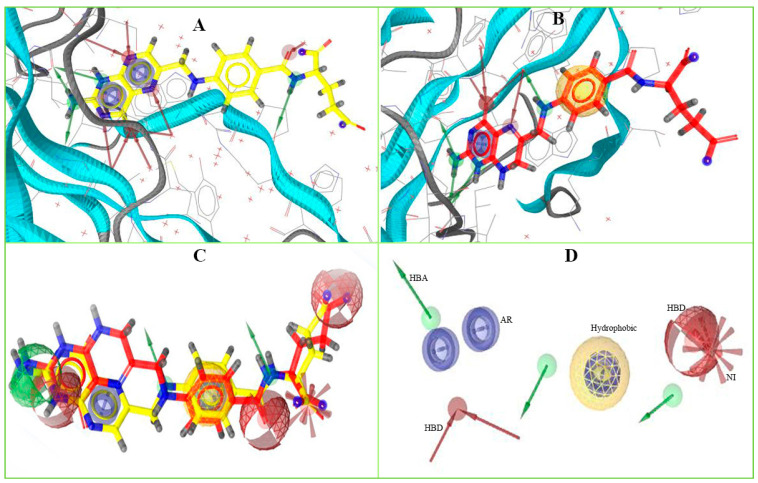
The overall pharmacophore model generated during the study. Herein, (**A**) pharmacophore features generated from 5VYH (PDB)-Folic Acid (yellow) complex interaction, (**B**) 6PXH (PDB)-Dihydrofolic Acid (red) complex interaction, (**C**) merge pharmacophore features, and (**D**) final pharmacophore features utilized for virtual screening. The hydrogen bond donor (HBD) features have shown in green, hydrogen bond acceptor (HBA) in red, negative ionizable area (NI) in red astricts, aromatic ring (AR) in blue, and hydrophobic (H) features in yellow color.

To identify novel scaffolds as a lead compound against MERS-CoV S1-NTD, two different pharmacophore models were generated based on the protein PDB ID: 5VYH and 6PXH in complex with folic acid (FOL409) and dihydro-folic acid (DHF428), respectively. The ligand (FOL409) in complex with the protein 5VYH generated a total of 14 pharmacophore features includes two aromatic ring (AR), eight hydrogen bond acceptor (HBA), and four hydrogen bond donor (HBD) features, where complex of DHF428 and 6PXH produced 10 pharmacophore features includes one AR, one hydrophobic (H), four HBA, and four HBD features shown in Figure 1A,B. This two-pharmacophore model was aligned and merged to interpolate overlapping features, which generated a total of 20 pharmacophore features including three AR, one H, two negative ionizable area (NI), 10 HBA, and four HBD features shown in Figure 1C. The overlapped and duplicate pharmacophore features from the aligned pharmacophore models have been removed to optimize and relaxed the geometrical confirmation of the model. After removing the duplicate pharmacophore features a total of 11 features includes three HBD, one H, three HBA, three AR, and one NI feature were selected for further study shown in Figure 1D. Exclusion volume coat generated during the pharmacophore modeling process has not been considered in the study.

### 2.2. Molecule Library Preparation

Virtual screening can be defined as a cheminformatics technology that utilizes different computational techniques to screen a large number of molecules and identify the structures of interest for biological assays [19]. The accuracy of a cheminformatics model depends on the data mining process that is related to database preparation. Therefore, to accurately mine the database, a total of 11,295 natural compounds have been retrieved from the Ambinter, and a library has prepared for virtual screening. The geometry of all the molecular structures has been optimized by conforming MMFF94 force field available at the LigandScout tool and a molecular library has been prepared [20]. The library prepared through the software has further utilized for the virtual screening process.

### 2.3. Active Compounds Identification and Decoy Set Generation

**Figure 2:**
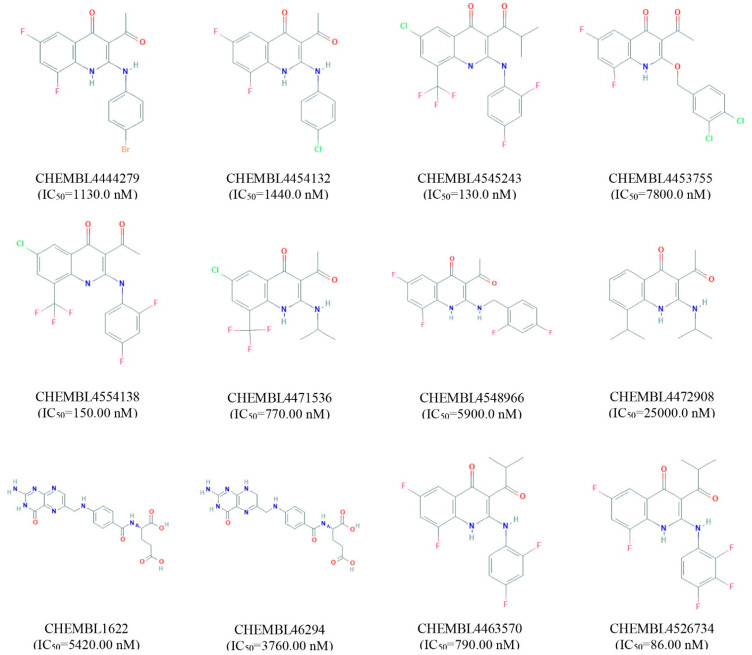
List of active compounds identified against MERS coronavirus S1-NTD protein. The IC_50_ value and correspondence ChEMBL identity for each compound has also been provided.

Validation of a pharmacophore model is essential before a large database screening process can provide reliable outcomes on a real-life project. The SBPM can be validated through known active compounds together with inactive compounds called “decoys”. Ideally, active compounds for model validation should be selected based on experimental data [19]. Therefore, 12 experimentally active compounds against MERS-CoV S protein have been identified and retrieved from the ChEMBL database. The active compounds have been selected based on their half maximal inhibitory concentration IC_50_ (nM) value shown in Figure 2. The active compounds then submitted into the DUDE-E decoy database and a total of 1326 decoys correspondence to active compounds has been retrieved. The geometry of the compounds has also been optimized by using the MMFF94 force field and converted into the LDB file format through LiganScout software.

### 2.4. Pharmacophore-Based Virtual Screening

In-silico virtual screening is a type of computational approach by which molecules with desired properties can retrieve structures with similar properties from large molecule libraries. During the drug design and development process, this technique helps to identify small molecules as hits and further optimization as lead candidates [21]. Furthermore, this process can help to reduce the assay-to-lead attrition rate that has excluded time and expensive experiments require during the drug design and development process. A specific 3D pharmacophoric pattern searching approach to screen large molecule libraries is now being considered as the next step in the drug design process. Therefore, the study utilized a 3D pharmacophore models-based virtual screening process to identify hit compounds against the targeted protein. The structure-based virtual screening process retrieved 32 active compounds as hits with a geometric fit score of 65.46 to 67.75, where the number of conformations generated during the screening was a minimum of eight and a maximum of 25 shown in Table S1.

### 2.5. Pharmacophore Model Performance Analysis

**Figure 3:**
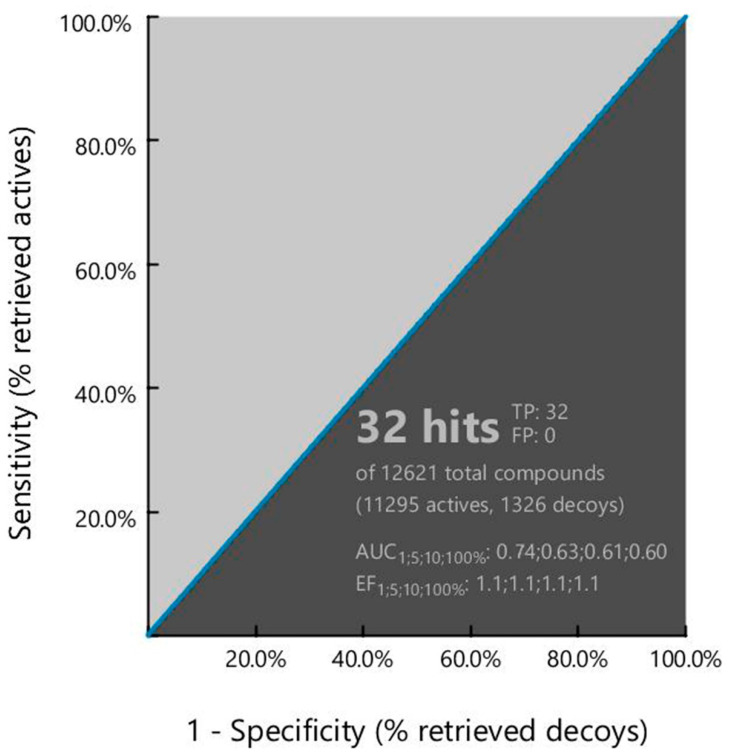
Showing the ROC curve generated during pharmacophore model-based virtual screening. The curve presents the relationship between sensitivity (true positive fraction to the *Y*-axis) and specificity (false positive fraction to the *x*-axis) for every possible cut-off.

To determine the performance of the pharmacophore model, the ROC curve generated during the virtual screening process has been analyzed. Receiver-operating characteristic (ROC) is a simple and useful graphical tool for evaluating the accuracy of a statistical model. The ROC curve in the virtual screening process provides information regarding the discrimination ability of the model from active to inactive (decoy) set [19]. The overall summary of the model accuracy can be calculated from the Area Under the Curve (AUC) that represents the degree of discrimination ability. The AUC value ranges between 0.0 to 1.0, where a value between 0 to 0.5 indicates random chance of discrimination, 0.51 to 0.7 indicates acceptable, 0.71 to 0.8 indicates good, and 0.81 to 1.0 indicates the excellence of the model [19]. The enrichment factor in the pharmacophore model provides an idea about the number of active compounds found from a specific model compared to hypothetically active compounds found from a randomly screened model. The EF factor can range from 1 to >100, where 1 indicates the number of randomly sorted molecules and >100 indicates the least number of compounds need to screen in vitro to find a large number of active compounds. The AUC and EF values found in the study were 0.74 and 1.1, respectively, indicating good discrimination ability and robustness of the pharmacophore model shown in Figure 3.

### 2.6. Binding Site Identification and Receptor Grid Generation

**Figure 4:**
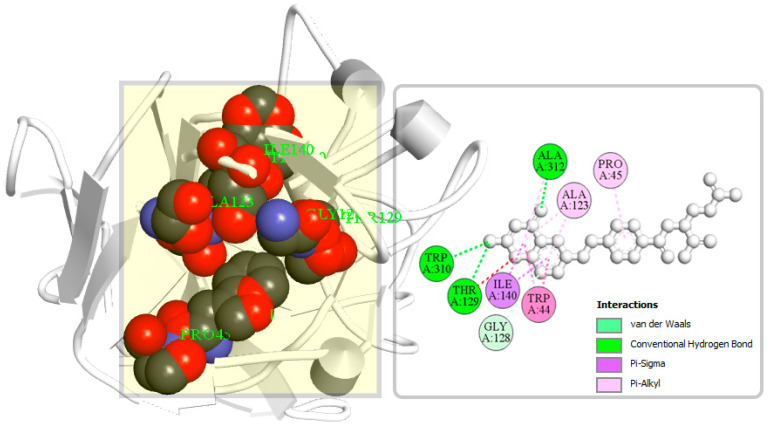
The binding site position of MERS-CoV S1-NTD identified from the protein–ligand complex (PDB ID: 5VYH) structure. Ball shape 3D representation of the binding site with the grid box shown on the left side in the figure, where 2D binding site position has also been represented on the right side of the figure.

A binding site can be defined as a specific amino acid (AA) residue in a protein to which ligands can binds and is fundamentally important for guiding drug design. Identification of the location of protein binding sites is essential during molecular docking simulation, which helps to generate enough contact points with the protein and significantly increases the docking efficiency [22]. Binding site is evolved to be optimized to bind a particular substrate, therefore the binding site of the protein has been identified in this study. Analysis of previously identified complex protein–ligands (PDB: 5VYH) interaction found eight binding site residues in the protein. The eight-binding site residues was resided at TRP44, PRO45, ALA123, GLY128, THR129, ILE140, TRP310, and ALA312 in the S1-NTD protein has been represented in a ball shape and shown in Figure 4.

PyRx is a grid-based docking program that requires the definition of receptor grid box size before initiating the molecular docking process. Grid box fixation before the molecular docking process helps to generate more reliable scoring to the ligand poses. Therefore, to identify more reliable ligand poses towards the protein, a receptor grid box with a dimension X = 30.69 (Å), Y = 33.36 (Å), and Z = 43.41(Å) has been generated based on previously identified binding residues position of the protein.

### 2.7. Molecular Docking Simulation

Molecular docking in CADD is an important technique that helps to determine the bound geometry and interaction between a small molecule and a protein at the atomic level. The technique has become an increasingly important tool for drug discovery due to the ability to screen large compound libraries [23]. The technique also helps to determine the behavior and predict how a protein (enzyme) interacts with small molecules (ligands) to the binding site of target proteins. To elucidate the ligand–receptor binding mechanism, a molecular docking simulation has been performed in this study. The 32 hits identified previously through the structure-based virtual screening process have been docked to the binding site of the MERS-CoV S1-NTD protein. The docking score found for the 32 hits has a range between −6.4 and −9.2 kcal/mol provided in Table S1. Based on the binding affinity top (10%), four compounds Amb6600135 (−9.2 kcal/mol), Amb23604132 (−9.1 kcal/mol), Amb23604659 (−8.6 kcal/mol), and Amb1153724 (−8.1 kcal/mol) with zero upper and lower RMSD value have been chosen for further evaluation listed in Table 1.

### 2.8. ADME Analysis

ADME properties of chemical compounds play an important role in the likelihood of success of a drug. Optimization of the ADME properties can reduce the pharmacokinetics-related failure in the clinical phases, which is difficult and challenging in the drug development and discovery process [24]. It has been found that early-stage evaluation of ADME can reduce the attrition rates during the clinical drug development phase. Therefore, the study utilized the SwissADME web tool for the early-stage evaluation of ADME properties for selected four compounds. The server evaluated the ADME properties of selected four (Amb6600135, Amb23604132, Amb23604659, and Amb1153724) compounds based on lipophilicity, solubility, pharmacokinetics, medicinal chemistry, and drug-likeness properties.

All the compounds have maintained an optimum pharmacokinetics property except the compounds Amb6600135, which has negative Log P_o/w_ value, active P-glycoprotein (P-GP) substrate (Figure S1) and violated the maximum Lipinski’s rule of five (RO5) listed in Table 2. On the other hand, the synthesis accessibility of the compound (Amb6600135) was higher (difficult to synthesize) than the other three compounds. Therefore, the compound has not been considered for further stages of evaluation.

### 2.9. Toxicity Test

Analysis of toxicity is an important and one of the main steps in drug design that helps to identify the harmful effects of chemical substances on humans, animals, plants, or the environment. Traditional assessment of compounds toxicity requires in vivo animal model, which is time-consuming, expensive, and subject to be ethical considerations [25]. Therefore, computer-aided in silico toxicity measurement of chemical substances can be considered useful in the drug design process. The study utilized the ProTox-II web server to identify the toxicity of the compound computationally, as it is not time-consuming, non-expensive, and requires no ethical considerations. The three compounds (Amb23604132, Amb23604659, and Amb1153724) selected previously through different screening process have been submitted in the ProTox-II web server that determines the acute toxicity, hepatotoxicity, cytotoxicity, carcinogenicity, and mutagenicity of the compounds listed in Table 3. All three compounds have shown no oral toxicity or organ toxicity effect.

### 2.10. Theoretical Calculation

#### Geometry Optimization

**Figure 5:**
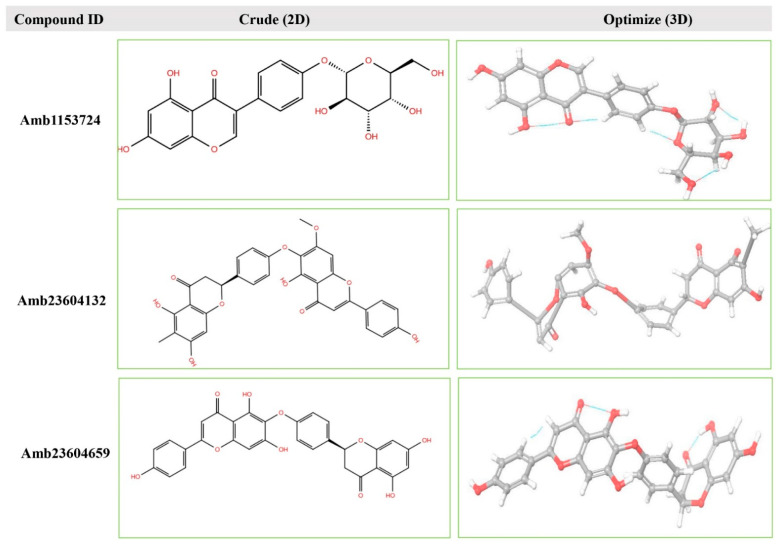
The 2D structures and 3D optimized molecular geometries of selected three compounds, Amb1153724, Amb23604132, and Amb23604659 calculated using B3LYP/6-31G(d,p) level of DFT calculations.

Geometry optimization is a quantum chemical technique used by most computational biologist, chemists, academics, and researchers to find the configuration of minimum energy with the most stable form of a chemical structure. It is a method of taking rough geometric approximations and making them as exact as possible [26]. The geometry with the lowest energy is the most stable because molecules with lowest energy state spontaneously decrease its energy by emitting. Therefore, the best optimized molecular geometry with the lowest energy value has been determined by using the default basis set 6-31G(d,p) in Jaguar. The 2D structures and 3D optimized geometries of the compounds Amb23604659, Amb23604132, and Amb1153724 have been plotted in Figure 5. Additionally, the bond angles, bond lengths (bohr, angstroms), and torsional angles optimized during the process have been provided in Supplementary text file format (renamed as Geometry). The optimized structure has been retrieved for further evaluation through molecular docking simulation.

### 2.11. Frontier Molecular Orbital HOMO/LUMO Calculation

**Figure 6:**
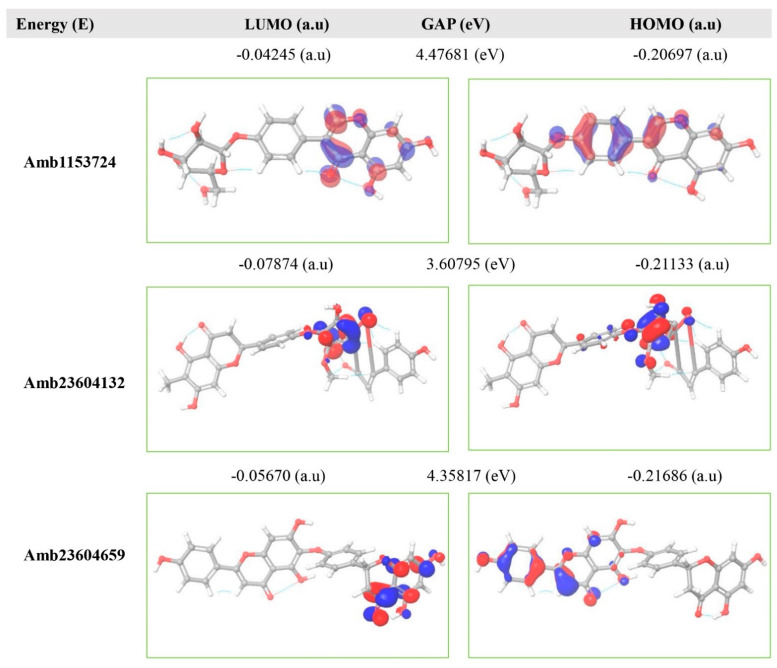
Representing the asymmetric HOMO, LUMO, and HOMO-LUMO gap energy for selected three natural compounds, where red is representing negative and blue represents positive phases of the molecular frontier orbital wave function.

The FMO is now significantly used in organic chemistry to explain the structure and reactivity of molecules. The theory can describe the electronic and optical properties of molecules by utilizing HOMO-LUMO bandgap energy [27]. The energy gap between the two orbitals HOMO and LUMO also helps to determine the sensitivity of atoms toward electrophilic and nucleophilic attacks, chemical kinetic stability, chemical hardness, and softness of a molecule [26]. The electrons localized from the HOMO orbital is most free to participate in the nucleophilic reaction, where the LUMO participates in the electrophilic reaction. A molecule with low HOMO-LUMO gap energy should have a high chemical reactivity and low kinetic stability that can be considered as a soft molecule. In this process, a molecule with a high frontier (HOMO-LUMO) orbital gap should have low chemical reactivity or bioactive and high kinetic stability due to the low probability of adding an electron to the high-energy LUMO. The molecules with a high FMO energy gap are energetically stable related to low chemical reactivity and high kinetic stability compared to a molecule having a low FMO energy gap [27]. Therefore, to evaluate the chemical reactivity and kinetic stability of the selected three compounds the HOMO, LUMO, and HOMO-LUMO, gap energy was calculated from equation (3) and shown in Figure 6, the hardness and softness of the molecules have also been calculated and listed in Table S2. The calculated FMO energy band gap values found for the compounds Amb1153724, Amb23604132, and Amb23604659 was 4.48 eV, 3.60 eV, and 4.35 eV, respectively, which was considerably higher, indicating kinetic stability and low chemical reactivity of the molecules.

### 2.12. Re-Docking, Interaction, and Pharmacophore Analysis

#### 2.12.1. Redocking Score

The re-docking process has been performed to identify the possible docking poses in a restricted area by using the previously obtained binding sites of the S1 protein. The geometry optimized structure has been docked and the score found for the selected three compounds Amb23604132, Amb23604659, and Amb1153724 were −10.2 kcal/mol, −9.5 kcal/mol, and −9.2 kcal/mol, which was better than the previously obtained binding score (Table 1). Therefore, it can be considered that the QM-based optimization of the compounds was effective for the selected three compounds.

#### 2.12.2. Protein–Ligands Interaction Interpretation

**Figure 7:**
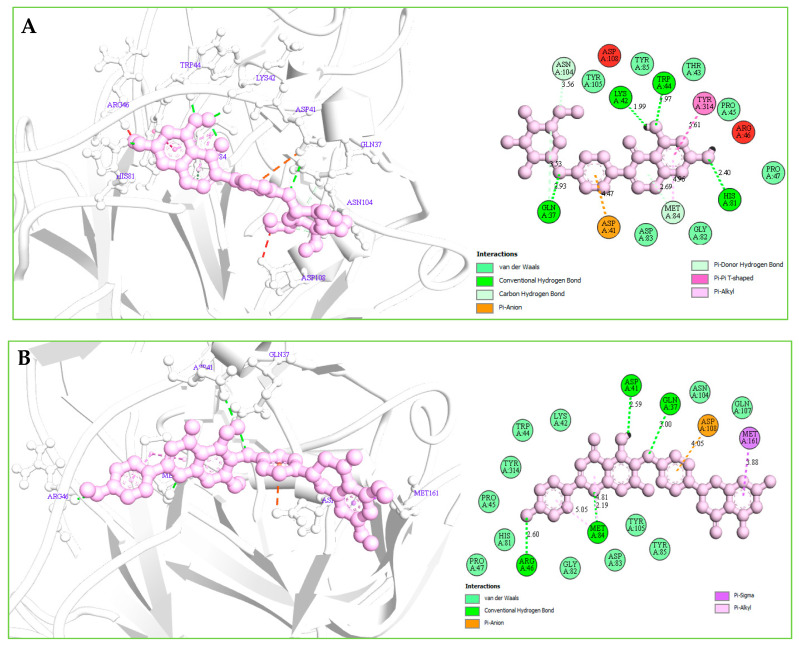

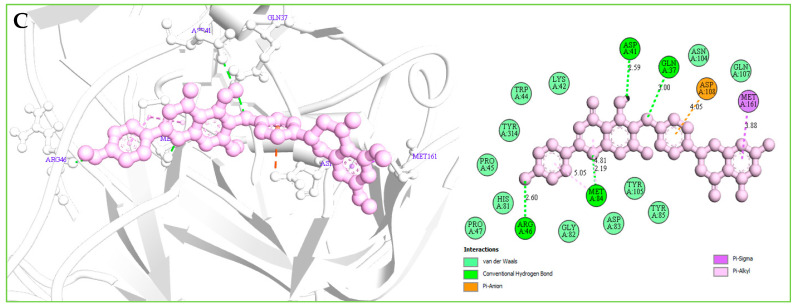
(**A**): The interaction between the MERS-CoV S1-NTD and Amb1153724 compounds. The 3D interaction has represented left side of the figure, where 2D interaction has depicted in right side of the figure accordingly. (**B**): The interaction between the MERS-CoV S1-NTD and Amb23604659 compounds. The 3D interaction has represented left side of the figure, where 2D interaction has depicted in right side of the figure accordingly. (**C**): The interaction between the MERS-CoV S1-NTD and Amb23604659 compounds. The 3D interaction has represented left side of the figure, where 2D interaction has been depicted in the right side of the figure accordingly.

Understanding the potential interactions between a protein–ligand complex is an important part of the field of drug discovery, which helps to identify hits to leads as a potential drug candidate. Interaction analysis also helps to navigate the position of small molecules in a protein and determine the behavior on biological networks. Accurate identification of protein–ligand interactions play a key role in drug development and disease treatment. Therefore, the interaction between the selected three compounds and desire S1-NTD protein has been analyzed by using the BIOVIA Discovery Studio Visualizer tools. Analysis of the complex structure identified different bonding interaction includes hydrogen bond (Conventional H-B, Carbon H-B, and Pi-Donor H-B), electrostatic (Pi-Anion), hydrophobic (Alkyl, Pi-Alkyl, Pi-Pi T-shaped, and Pi-Sigma) between the protein and ligand listed in Table 4 and depicted in Figure 7.

Complex structure analysis of Amb1153724 found a total of eleven bonding interaction including seven hydrogen bonds (four conventional H-B, two carbon H-B, and one Pi-Donor H-B), one electrostatic (Pi-Anion), and three hydrophobic (two Pi-Alkyl, one Pi-Pi T-shaped) bonds to the different binding site residues of the protein, which have bonds distance range between minimum 1.99 Å to maximum 5.61 Å shown in Figure 7A and listed in Table 4.

The compounds Amb23604659 have been found to form a total of ten bands including five hydrogen bonds (four conventional H-B and one carbon H-B), one electrostatic (Pi-Anion), and four hydrophobic bonds (one Alkyl, two Pi-Alkyl, one Pi-Pi T-shaped) with the protein in different residual position having a distance between 2.0 Å to 5.10 Å shown in Figure 7B.

For the compound Amb23604659, a total of seven bonds have been found to form, which have a bond distance range between a minimum of 2.0 Å to a maximum of 5.10 Å. The compounds formed four conventional hydrogen bonds, an electrostatic (Pi-Anion) bond, and one Pi-Sigma hydrophobic bond with the S1 protein shown in Figure 7C.

#### 2.12.3. Pharmacophore Features Analysis

Pharmacophore features of a compound play an important role during the molecular recognition process of targeted biological macromolecules. The pharmacophore of a compound can be described based on the H, AR, HBA or HBD, PI, NI features. These features can derive from the ligand or projected points believe to reside in the protein that helps to identify and design a new drug for the treatment of a selected disease.

**Figure 8:**
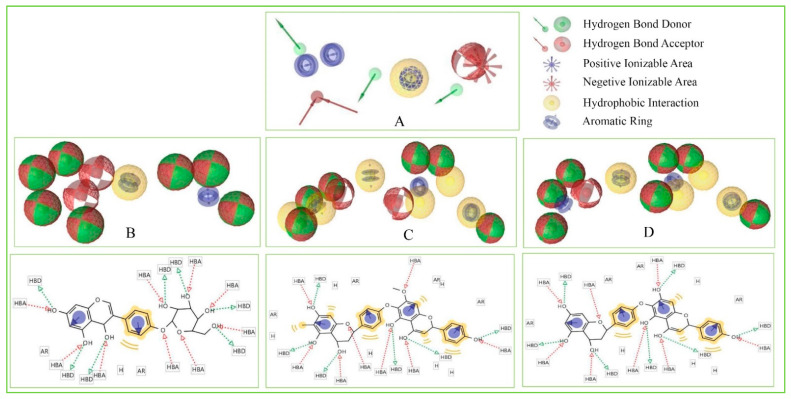
Showing the pharmacophore features of selected three compounds in 3D and 2D format along with the query features that have previously been used to screen the compounds. Herein, the figure representing (**A**) Query pharmacophore features and features generated from the compounds (**B**) Amb1153724, (**C**) Amb23604132, and (**D**) Amb23604659 during the screening process.

These features retain the necessary geometric arrangement of atoms requires to producing a specific biological response. Therefore, the pharmacophore features of the selected three compounds include Amb23604659, Amb23604132, and Amb1153724 have been analyzed and compared with the query pharmacophore features shown in Figure 8. The 14 pharmacophore features used to screen the compounds generated 32 hits, which has further screened through the different screening process and identified three compounds as potential drug candidates. Each of the compounds generated 25 confirmations that have better pharmacophore properties than the query pharmacophore features. Therefore, the selected compounds should be effective to our target protein.

### 2.13. MD Simulations Analysis

MD simulations help to determine the physical movements of atoms and molecules by simulating the system at an atomistic scale. The invaluable technique for observing biomolecular structure and dynamics has expanded dramatically in recent years. The MD simulation offers a great and distinct approach to investigate the stability of a ligand to a targeted macromolecule. Therefore, to identify the stability of the selected three compounds with the desired protein, a 200 ns MD simulation has been performed for each complex structure and described based on the RMSD, RMSF, and protein–ligand contact mapping.

#### 2.13.1. RMSD Analysis

RMSD of a protein–ligand complex system helps to determine the average distance generated through the dislocation of an elected atom during a specific time compared to a mentioned time [4]. RMSD of the selected three compounds has been observed to identify the changes in protein structure as compared to the starting point. It also helps to determine the equilibration state of the protein determined from the flattening of the RMSD curve. Initially, the protein frames and the reference frame backbone were aligned during the MD simulation and then the RMSD of the system has been calculated based on the atom selection. The complex system with a time frame x should have the RMSD that can be calculated from the following Equation (1).

(1) $$RMSD_{x}=\sqrt{\frac{1}{N}}\overset{N}{\underset{i=1}{\sum }}{\left({r^{\prime }}_{i}\left(t_{x}\right))-r_{i}\left(t_{ref}\right)\right)}^{2}$$

Here, the *RMSD_x_* is the calculation of RMSD for the specific number of frames, *N* is the number of selected atoms; *t_ref_* is the reference or mentioned time, and *r′* is the selected atom in the frame *x* after superimposing on the reference frame, *t_x_* is the recording intervals.

**Figure 9:**
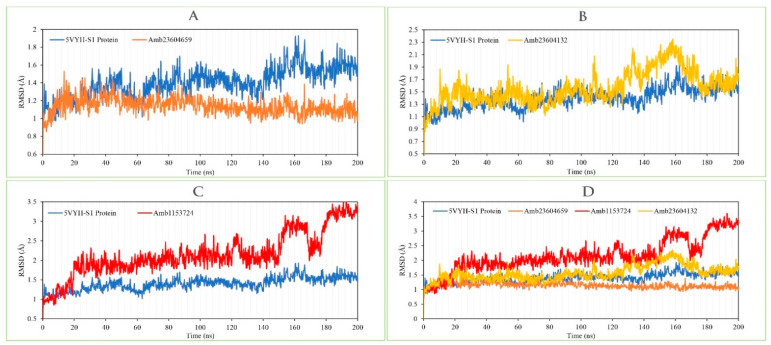
Depicted the RMSD values extracted from the Cα atoms of the selected three compounds in complex with the MERS-CoV S1-NTD protein. Herein, showing the RMSD of S1-NTD (Blue) in complex with the compounds (**A**) Amb23604659 (orange), (**B**) Amb23604132 (Yellow), and (**C**) Amb1153724 (Red), where (**D**) representing all the compounds and protein RMSD together.

The RMSD of the selected three compounds and the protein has been analyzed to determine the system has equilibrated or not. The RMSD of selected three compounds Amb23604659, Amb23604132, and Amb1153724 complex structure has been compared with the native S1 protein structure to observe the changes of the order shown in Figure 9. The RMSD for all the compounds was in a range between 1.0 Å to 2.5 Å that was perfectly acceptable compared to the structure of the native protein. The highest fluctuations (<3.0 Å) found for the compounds Amb1153724 between 185 ns to 200 ns simulation time and gradually stabilize, however, indicate that the compound has undergone a small conformational change during the simulation. It has been found that the simulation was converged between 20 ns to 160 ns for all the compounds and the RMSD values have been stabilized around a fixed value within the time. The fluctuations for all the selected compounds towards the end of the simulation were around some thermal average structure. Therefore, the selected compounds can be considered as stable to the targeted protein. Additionally, the RMSD for all the selected three ligands was observed to show how stable the ligand was concerning the desired protein and its binding site (Figure S3). The values observed for the ligand were closer than the RMSD of the S1-NTD protein, then it has been considered that the ligand will not be diffused away from its initial binding site.

#### 2.13.2. RMSF Analysis

The RMSF is important to observe the local changes of a protein that helps to measure the displacement of a specific atom compared to the reference structure by calculating the average change observe over the number of atoms [19,22]. This is a numerical calculation like RMSD useful for characterizing a protein, which can determine the residue flexibility and fluctuation during the simulation. The RMSF for residue *i* has been calculated from the following Equation (2).

(2) $$RMSF_{i}=\sqrt{\frac{1}{T}}\overset{T}{\underset{t=1}{\sum }}<{\left({r^{\prime }}_{i}\left(t\right))-r_{i}\left(t_{ref}\right)\right)}^{2}>$$

where *T* is the overall trajectory time, *r_i_* is the residue location, *t_ref_* is the reference time, *r’* is the location of atoms in residue *i* after aligned on the reference, and the angle brackets (< >) are the average of the square distance.

**Figure 10:**
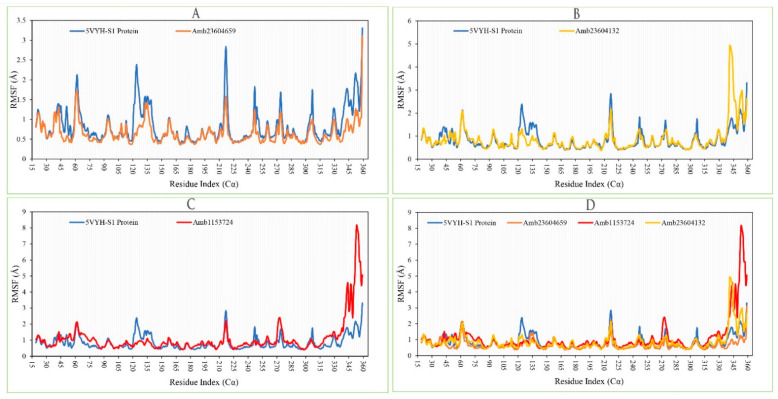
Showing the RMSF values extracted from the Cα atoms of the selected three complex structures. Herein, showing the RMSF of S1-NTD protein (Blue) in complex with the compounds (**A**) Amb23604659 (orange), (**B**) Amb23604132 (Yellow), and (**C**) Amb1153724 (Red), where (**D**) representing all the compounds and protein RMSF together.

The RMSF of the selected three complex structures has been analyzed to measure the displacement of a particular atom during the simulation. The RMSF of the selected three complex structures has been compared with the native S1-NTD protein structure to observe the atomic changes of the order shown in Figure 10. On this figure, the peaks indicate the protein fluctuation of the Amb23604659, Amb23604132, and Amb1153724 complex structure, which found minimal between 30 to 340 AA residue of the most rigid secondary structure elements includes alpha-helices and beta-strands. The highest fluctuation founds for all the three compounds before 30 AA and after 340 AA residue due to the location of the N- and C-terminal domain. Therefore, it can be considered that the displacement of a particular atom or a group of atoms will be lower in a real-life environment for all the selected three compounds.

#### 2.13.3. Protein–Ligands Contact Analysis

**Figure 11:**
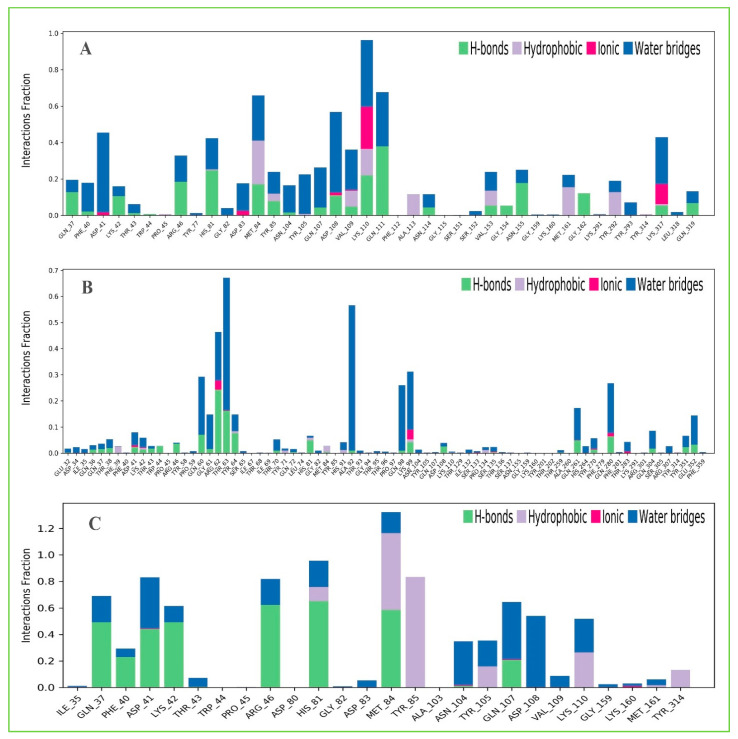
The stacked bar charts showing the protein–ligands interactions found during the 200 ns simulation run. Herein, showing the selected three ligands (**A**) Amb23604659, (**B**) Amb23604132, and (**C**) Amb1153724 contact map with the desire S1-NTD protein.

Protein interactions with the selected three ligands Amb23604659, Amb23604132, and Amb1153724 have been monitored throughout the SID. The hydrogen bonds, hydrophobic, ionic, and water bridge interactions found during the MD simulation have been observed and shown in the stacked bar charts (Figure 11). The different types of bonding interaction play a significant role in ligand binding to the targeted protein, where hydrogen-bonding properties in drug design play an important role to influence drug specificity, metabolization, and adsorption. The hydrogen bonding interaction found for all three compounds was observable until the last AA residue during the simulation. For all the complex structures, it has also been found to form multiple interactions (hydrogen bonds, hydrophobic, ionic, and water bridges) at the same residue position of the protein with the ligand represented by a darker shade of orange, according to the scale to the right of the plot (Figure S2). The compound Amb23604659 generated multiple (more than two) interactions at ASP41, HIS81, MET84, TYR85, ASP108, VAL109, and LYS110 residues with an interaction fraction (IF) value 0.50, 0.45, 0.75, 0.25, 0.40, and 0.98, respectively indicating that 50%, 45%, 75%, 25%, 40%, and 98% of the simulation time the specific interaction is maintained by the multiple contacts of the same subtype with the ligand accordingly. The compound Amb23604132 formed multiple interaction at ASP41 (0.1), LYS42 (0.09), ARG62 (0.5), THR63 (0.68), HIS81 (0.1), LYS99 (0.38), GLN261 (0.2), TYR270 (0.05), GLN 280 (0.3) residues maintained by 10%, 9%, 50%, 68%, 10%, 38%, 20%, 5%, and 30% simulation time accordingly. In the case of the compound Amb1153724, it has found to form multiple interactions at the position of ASP41 (0.85), HIS81 (0.99), MET84 (1.3), and GLN107 (0.58) suggests that 85%, 99%, 130%, and 58% of the simulation time the specific interaction is maintained and helped to make a stable binding with the desired protein.

#### 2.13.4. Ligand Properties Analysis

Ligand properties were analyzed to evaluate the stabilities of the selected three compounds Amb23604659, Amb23604132, and Amb1153724 under the MD simulation. The ligands properties were analyzed based on the RMSD of the ligands, Radius of Gyration (rGyr), Intramolecular Hydrogen Bonds (intraHB), Molecular Surface Area (MolSA), Solvent Accessible Surface Area (SASA), and Polar Surface Area (PSA), which found favorable for all the three compounds shown in Figure S3. Additionally, the selected three ligands and the co-crystal ligand (folic acid) RMSD have combined analysis and been compared, which has been provided in Figure S4.

### 2.14. MM/GBSA Analysis

**Figure 12:**
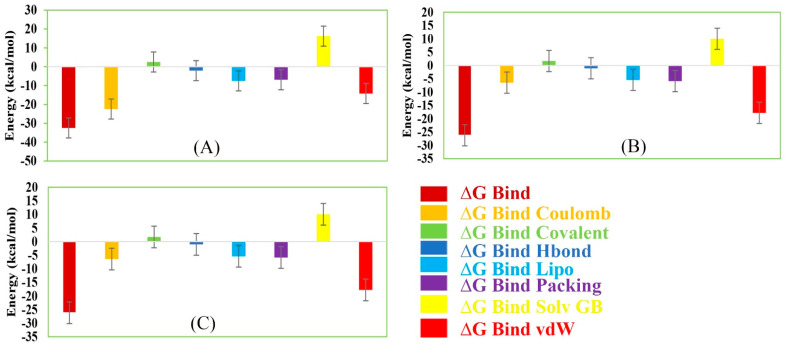
Representing different energy components and net MM/GBSA binding free energy (kcal/mol) with standard deviation values for extracted snapshots of MERS-CoV S1-NTD protein in complex with selected compounds, i.e., (**A**) Amb1153724, (**B**) Amb23604132, and (**C**) Amb23604659 from respective 200 ns MD simulation trajectories.

The MM/GBSA methods have been used to calculate the end-point binding free energy of the protein–ligand complex. The MM/GBSA of the complex system has been calculated from the single trajectory collected from the respective 200 ns simulation (Table S3). Analysis of MM/GBSA found higher net negative binding free energy values for the selected compounds Amb1153724, Amb23604132, and Amb23604659 in complex with MERS-CoV S1-NTD protein (Figure 12). The complex analysis of MM/GBSA found −32.47 kcal/mol, −24.75 kcal/mol, and −26.18 kcal/mol binding free energy for the compounds Amb1153724, Amb23604132, and Amb23604659, respectively, at the last stage of the MD simulation. Therefore, the screened compounds will be able to maintain a durable interaction with the desired protein. Additionally, analysis of physico-chemical components for the selected three compounds revealed a significant contribution of G_Bind Coulomb_ (Coulomb energy) and G_Bind vdW_ (Van der Waals interaction energy) shown in Figure 11. From the above result it can be suggested that the selected three compounds can maintain a long-term interaction with the MERS-CoV S1-NTD protein binding site and result in inhibition of the desired protein.

## 3. Discussion

Since the emergence of MERS-CoV in 2012, necessary steps to revoking the infection caused by the pathogen have become a major research focus. However, to date, no effective anti-viral drug candidates against the zoonotic pathogen have developed yet. It has been found that the distinctive NTD of the virus S1 subunit functioning as a RBD, which plays an important role to determine the host range resulting in cross-species infection [9]. Therefore, the study aimed to inhibit the function of S1-NTD of the virus to identify a novel and effective antiviral drug candidate against MERS-CoV infections.

In this study, we first identified the available experimental protein structure of MERS-CoV S1-NTD in complex with different inhibitory compounds from the protein databank. The MERS-CoV S1-NTD having a complex structure was used to generate an SBPM to the active site cavity of the protein. All the individual pharmacophore models generated from the complex structure have merged, and a combined final pharmacophore model has been generated to screen 11,295 natural compounds collected from the Ambinter natural compounds database. The pharmacophore model has validated using 12 experimentally known active compounds with their correspondence 1326 decoy compounds, where the AUC under the ROC curve indicated good discrimination ability of the model. The validated pharmacophore model has been utilized for the virtual screening process and retrieved 32 compounds as hits, which has been further screened through molecular docking simulation methods. Based on the molecular docking score, the top four compounds having a binding score of >8.0 kcal/mol have been chosen for further validation.

The selected four compounds Amb6600135, Amb23604132, Amb23604659, and Amb1153724 have been evaluated based on the ADME properties, where all compounds except the compound Amb6600135 have shown a good value of the ADME properties. The compound Amb6600135 disobeyed maximum (three) Lipinski’s rule of five, on the other hand, the P-GP efflux pump was active of the compound (Figure S1), therefore the compound has skipped for further evaluation. The compound with good ADME properties has been further evaluated through the toxicity properties to measure the harmful effect on humans or animals. Analysis of toxicity found no or low toxicity of the selected three compounds.

To investigate and optimize the geometry of the compounds a computational DFT-based QM simulation has been performed. The geometry optimized through the DFT has been retrieved and re-docked with the desired protein, which exhibited substantial docking energy >−9.00 kcal/mol. The FMO based HOMO-LUMO energy gap was also calculated to evaluate the chemical reactivity of the compounds. The HOMO-LUMO gap energy found for all the compounds was high >3.50 eV which confirms the low reactivity correspondence to the bioactivity of the compounds.

The geometry optimized re-docked complex structure have been stimulated again by the MD simulation approach to identify the stability of the compounds to the binding site of the protein. The 200 ns simulation trajectories have been retrieved, and analysis based on the RMSD, RMSF, protein–ligand contact mapping, and ligand torsion properties (Figure S5) that confirm the stability of the compounds to the binding sites of the protein. Additionally, the MM/GBSA calculated from the single trajectory found a high ΔG_bind_ value, indicating the stability of the selected protein–ligand complex for long-term simulation.

## 4. Conclusions

To the best of our knowledge, this study offers the first compressive in-silico approaches to identify potential natural antiviral drug candidates against MERS-CoV S1-NTD. An integrative structure-based pharmacophore modeling, virtual screening, molecular docking, ADMET, QM calculation, MD simulation, and MM/GBSA approaches revealed Taiwanhomoflavone B, 2,3-Dihydrohinokiflavone, and Sophoricoside as potential drug candidates that will help to inhibit the activity of the S1-NTD of the virus. Further evaluation through different lab-based experiment techniques can help to determine the activity of the compound that will provide alternatives for MERS-CoV immunotherapy.

## 5. Materials and Methods

### 5.1. Pharmacophore Modeling

The crystallographic structure of MERS coronavirus S1-NTD submitted between 2012 to 2021 was searched in Protein Data Bank (PDB). A total of 16 S1-NTD protein structures generated through X-ray crystallographic method were identified from the PDB and filtered based on the protein resolution, having a range between 1 to 2.5 Å. After filtration six protein structures were retrieved, where two (PDB ID: 5VYH and 6PXH) protein structures were in a complex with potential inhibitors and selected as an input to generate a SBPM. This two-crystal structure corresponding to MERS coronavirus S1-NTD protein is complexed with its potential inhibitor Folic Acid (FOL409: A) and Dihydrofolic Acid (DHF428: A), were chosen to generate the pharmacophore model [10,13]. The protein structure in complex with different inhibitors was optimized by using the MMFF94 force field available at LigandScout 4.4 software [20]. The LigandScout tools were also used to generate and analyze the pharmacophore features originated from the two selected crystallographic structures based on protein–ligands interaction. Initially, two separate pharmacophore models were developed by using the protein 5VYH and 6PXH in complex with Folic Acid and Dihydrofolic Acid, respectively. The pharmacophore features generated from the complex structure were centrally coordinated for alignment perspectives. After center coordinates of all the structures, a final pharmacophore model was created by using the align and merge pharmacophore features option available at the LigandScout tool. The software generated a combined pharmacophore model and provided a fit value by aligning the molecule on the pharmacophore model. The pharmacophore features observed in the study were described based on Hydrogen Bond Donor (HBD), Hydrogen Bond Acceptor (HBA), Positive Ionizable Area (PI), Negative Ionizable Area (NI), Hydrophobic Interactions (H), and Aromatic Ring (AR) features.

### 5.2. Molecule Library Preparation

Ambinter (www.ambinter.com) is a brand and worldwide supplier of advanced chemicals that supporting the scientific community by providing active compounds for drug discovery. The Ambinter database contains over 36 million molecules including screening molecules as well as a large collection of natural compounds. Therefore, the database contains a targeted library of SARS-CoV-2 has retrieved for the further screening process. LigandScout can screen single or multi-conformational compounds from a large database for drug design and discovery. The tool can recognize and screen molecular libraries having the proprietary LDB as a file format. Hence, it is important to convert the compounds library into the LDB file format before the virtual screening. In this study, the LigandScout tools have been utilized to prepare the molecular library.

### 5.3. Active Compounds Identification and Decoy Set Generation

Experimentally validated active compounds against MERS coronavirus S1-NTD have been identified from the ChEMBL database (https://www.ebi.ac.uk/chembl, accessed on 03 April 2021) [28]. The active compounds identified from the ChEMBL database have been submitted to DUDE-E (Database of Useful Decoys: Enhanced) decoy database available at (http://dude.docking.org/, accessed on 03 April 2021) [29]. The DUDE-E decoy database identified and generated a decoys compounds list correspondence to the active compounds. The decoy compounds have been retrieved and converted into LDB file format by using the LigandScout tool for the validation of the pharmacophore model.

### 5.4. Model Performance Analysis

To determine the performance and ability of discrimination between an active set from a decoy set a receiver operating characteristics (ROC) curve has been developed in this study. From the ROC curves, the Area under (AUC) the ROC Curve has been evaluated, which helps to measure the 2D area underneath the entire ROC curve. The ROC curve also helps to evaluate the Enrichment Factor (EF) of the pharmacophore model.

### 5.5. Virtual Screening

In-silico 3D pharmacophore-based virtual screening of the molecule libraries has been performed by using the LigandScout virtual screening tools. The final pharmacophore model generated in this study has been utilized as filter criteria for the database screening process. During the multitude pharmacophore screening processes, the parameters pharmacophore-fit has been chosen as the scoring function, match all query features as the screening mode, and first matching confirmation as the retrieval mode, where a maximum of five pharmacophore features have been omitted. The excluded volume clashes generated during the pharmacophore modeling have not been checked in the screening process. Before running the pharmacophore-based virtual screening process the natural compounds database were marked as active, where decoy compounds have been marked as inactive databases. After finishing the screening process, hit compounds with the number of confirmations and geometric fit scores were investigated for further evaluation.

### 5.6. Protein and Ligands Preparation

The crystal structure of MERS-CoV S1-NTD has been retrieved from the RCSB (www.rcsb.org, accessed on 03 April 2021) protein data bank (PDB ID: 5VYH) consisting of 343 amino acids (AA) length with a resolution value of 2.00 Å [13]. The S1-NTD protein was prepared by removing water, metal ions, and cofactors from the complex structure. The nonpolar hydrogen atoms were merged, polar hydrogen atoms were added, and Gasteiger charges were calculated for the protein [19]. The hits generated during pharmacophore-based virtual screening have been retrieved and prepared by adding gasteiger charges and AD4 atom types to the molecules. The non-polar hydrogens were merged, and aromatic carbons were detected to setting up the ‘torsion tree’ of the molecules by using AutoDockTools and were saved in PDBQT format for the further screening process.

### 5.7. Binding Site Identification and Grid Box Generation

Binding sites can be identified through the analysis of similar pockets from known protein–ligands interaction. The known and experimental validated S1-NTD protein structure in complex with the ligand folic acid was retrieved from the PDB (PDB ID: 5VYH) and the binding site of the protein has been analyzed through BIOVIA Discovery Studio Visualizer v19.1 (BIOVIA). The binding site determined from the complex structure has been utilized for the receptor grid generation during the molecular docking simulation by using the PyRx virtual screening tool.

### 5.8. Molecular Docking Simulation

To identify the best hit candidates against the desired protein, a molecular docking simulation has been performed by using the PyRx tool [30]. PyRx is an open-source virtual screening tool that includes both AutoDock 4 and AutoDock Vina as a docking wizard which can screen a large compounds database against a specific biological targeted macromolecule. The AutoDock Vina wizard with default configuration parameters of PyRx has been used for molecular docking simulation. The top 10% compounds, having the highest binding affinity (kcal/mol) to the desired protein, have been chosen for further evaluation.

### 5.9. ADME Analysis

In the early stage of the drug design and development process assessment of ADME, it is necessary to understand the safety and efficacy of a drug candidate that will be processed by a living organism. The ADME properties describe pharmacokinetics behavior and the movement of drugs into, though, and out of the body. Traditionally, the ADME properties were evaluated at the last stage of the drug discovery process, but in-silico tools can be predicted the properties at the early stages of the drug design process and help to optimize the pharmacodynamic response. To evaluate and understand the pharmacodynamic response of selected drug candidates, the SwissADME (http://www.swissadme.ch, accessed on 03 April 2021) web tool has been used in this study [24]. The freely accessible web server helps to predict the physicochemical, pharmacokinetics, and drug-likeness properties of the selected drug candidates.

### 5.10. Toxicity Test

Toxicity testing in the drug design and development process is essential to evaluate the compound’s toxic properties and the dose level requirements for the treatment of a specific disease. The toxicity profile of drug candidates gives an idea about the health and environmental risks and safety/toxicity of a chemical’s substances. Nowadays, computer-aided in-silico toxicity testing is playing an important role in the assessment of compounds toxicity more accurately without using the experimental animal models. Therefore, to evaluate the early-stage toxicity of the selected drug candidates ProTox-II (http://tox.charite.de/protox_II, accessed on 03 April 2021) webserver has been used, which helps to determine acute toxicity, hepatotoxicity, cytotoxicity, carcinogenicity, mutagenicity, and immunotoxicity of the selected compounds [25].

### 5.11. Quantum Mechanics (QM)-Based Calculation

Conformation analysis of a ligand to the binding site of a protein is an essential part to identify potential active conformation, binding affinity, and strain discipline associated with the binding mechanism. This type of binding possess can be achieved through the calculation of minimum energy conformation and structural optimization, which is dependent on the solution phase and associated gas-phase energy. The classical molecular mechanics (MM) process is unable to describe the process properly due to the presentation of metal ions in a ligand–protein complex system [31]. In the last few years, QM-based calculations have helped to enhance the scoring functions that can describe the electronic structure, electronic changes, and system-specific charges during a reaction of a molecular system. Interestingly, more than 80–90% of all QM-based calculations are nowadays solve depend on density functional theory (DFT). Therefore, this study performed the DFT methods-based QM calculations of selected three compounds. Initially, the bond lengths, bond angles, and dihedral angles for potential compounds were optimized, then the DFT of the compounds has been calculated by using the Schrödinger Jaguar version 10.9 [27]. Calculation of DFT has been performed by utilizing a mix of conventional functionals Becke’s three parameters with Lee-Yang-Parr functionals (B3LYP) and a dispersion correction energy term D3 combinedly known as B3LYP-D3. The conventional mix functionals B3LYP-D3 has been chosen in this study to not alter the wavefunction or any other molecular property directly, and 6-31G**, also known as 6-31G (d, p), has been chosen as a basis set to represent the electronic wave function of the molecules.

### 5.12. Frontier Molecular Orbital HOMO/LUMO Calculation

The highest energy occupied molecular orbital (HOMO) and lowest energy unoccupied molecular orbital (LUMO) are central to the frontier molecular orbital (FMO) theory or Fukui functions developed by Kenichi Fukui in the 1950s. The FMO of a molecule is the “frontier” of an electron that helps to determine the energy difference between two orbitals HOMO and LUMO. HOMO is mainly an electron donor (nucleophilic) and LUMO is an electron acceptor (electrophilic) in nature and the interaction between electron donor and electron acceptor pair can dominate other chemical reactivity of a molecule [32]. During the electrophilic-nucleophilic reaction, electrons from the HOMO jump to the LUMO and produce an energy difference between two molecular orbitals. The energy difference between two molecular orbitals is known as the HOMO-LUMO gap, which can explain the photochemistry and the strength and stability of transition metal complexes of organic molecules. To understand the sensitivity of atoms toward electrophilic and nucleophilic attacks, the HOMO and LUMO energy were calculated by using the Schrödinger Jaguar version 10.9 [27], and the energy difference between two molecular orbital HOMO-LUMO gaps was calculated from the following Equation (3).

(3) $$\Delta E_{\left(gap\right)}=E_{LUMO}-E_{HOMO}$$

where, ΔE is the HOMO-LUMO gaps, $E_{LUMO}$ is the lowest energy unoccupied molecular orbital energy, and $E_{HOMO}$ is the highest energy occupied molecular orbital energy.

### 5.13. Re-Docking and Interaction Analysis

Geometry optimized through the DFT-based QM method of selected three compounds has been retrieved and docked again to the same binding site of the MERS-COV S1-NTD. A rigid molecule docking was conducted in this study due to rigid molecules that are not change their spatial shape during the docking process. The docking was performed through the PyRx tools AutoDock vina by using the default parameter as a setting [23]. Herein, the best binding poses with the lowest root-mean-square deviation (RMSD) have been selected for binding interaction analysis. The complex protein–ligand interaction has been analyzed through BIOVIA Discovery Studio tools.

### 5.14. MD Simulation

To analyze the physical movements and behavior of the selected compounds in the macromolecular environment, the protein–ligand complex structure obtained from the re-docking studies was evaluated through 200 ns MD simulations. The physical motions of atoms in the protein molecules have been observed through the Desmond module of Schrödinger (Release 2020-3) under a Linux environment [19]. Initially, the predefined simple point-charge (SPC) water model has been used to solvate the complex system for obtaining the correct density and dielectric permittivity of water. The boundary condition selected in this study was orthorhombic (box shape), and a buffer box has been chosen as a calculation method with a box distance of 15 Å. To obtain and maintain a salt concentration of 0.15 M, the system has been neutralized by adding Na+ and Cl- ions. The isothermal-isobaric (NPT) ensemble has been performed at constant pressure (1.01325 bar) and temperature (300 K) with an energy value of 1.2. The atomic movement of the molecules has been recorded for every 2 ps recording interval and OPLS-2005 is set as a force field to obtain the trajectory as an output for 200 ns simulation.

#### Analysis of MD Trajectory

The simulation snapshots for each atomic movement have been recorded for 2 PS intervals were rendered by using Schrödinger maestro interface v9.5. The simulation event has been analyzed through the Simulation Interaction Diagram (SID) available at the Schrödinger package. From the trajectory output, root-mean-square fluctuation (RMSF), RMSD, and protein–ligand contacts (P–L contact) have also been analyzed.

### 5.15. End-Point Binding Free Energy Calculation with MM/GBSA

MM/GBSA are nowadays getting more popularity for estimating ligand-binding affinities in many systems. They are typically based on the MD simulations of the receptor-ligand complex, which is more accurate than most scoring functions of molecular docking and computationally less demanding to alchemical-free energy methods [33]. Therefore, to estimate ligand-binding free energy (ΔG_bind_) of the selected three compounds to the S1-NTD protein, the MM/GBSA methods have been performed by using the using Prime MM/GBSA module in the Schrödinger-Maestro package [26].

## Figures and Tables

**Table 1:** List of the top four compounds and their chemical name, molecular formula, binding affinity (kcal/mol), and pharmacophore fit score.

Ambinter ID	Molecule Name	Formula	Structure	Binding Affinity (kcal/mol)	Pharmacophore Fit Score
**Amb6600135**	Nicotinamide adenine dinucleotide	C_21_H_28_N_7_O_14_P_2+_	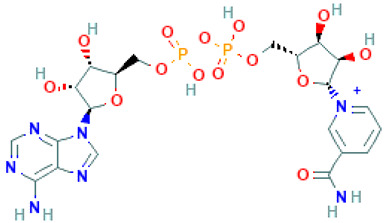	−9.2	66.6
**Amb23604132**	Taiwanhomoflavone B	C_32_H_24_O_10_	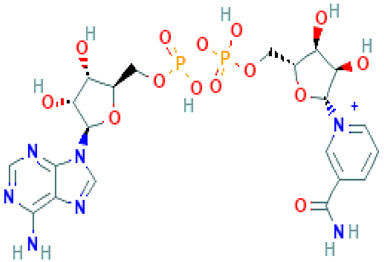	−9.1	65.48
**Amb23604659**	2,3-Dihydrohinokiflavone	C_30_H_20_O_10_	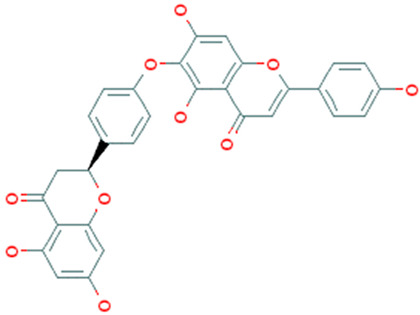	−8.6	65.55
**Amb1153724**	Sophoricoside	C_21_H_20_O_10_	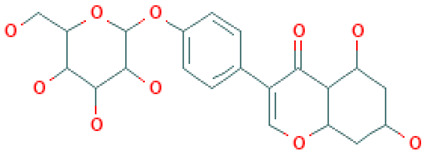	−8.1	66.64

**Table 2:** List of pharmacokinetics (ADME) properties includes physicochemical properties, lipophilicity, water-solubility, drug-likeness, and medicinal chemistry of selected four compounds.

Properties		Amb6600135	Amb23604132	Amb23604659	Amb1153724
Physico-chemical Properties	MW (g/mol)	664.43	568.53	540.47	432.38
	Heavy atoms	44	42	40	31
	Aro. atoms	15	28	28	16
	Rotatable bonds	11	5	4	4
	H-bond acceptors	17	10	10	10
	H-bond donors	8	4	5	6
	TPSA (Å^2^)	337.88	155.89	166.89	170.05
Lipophilicity	Log P_o/w_ (Cons)	-5.39	4.37	3.70	0.45
Water Solubility	Log S (ESOL)	High	Soluble	Soluble	Moderate
Pharmacokinetics	GI absorption	Low	Moderate	Moderate	Low
	BBB permeant	No	No	No	No
	P-GP substrate	Yes	No	No	No
Drug likeness	Lipinski violations	3	1	1	1
Medi. Chemistry	Synth. accessibility	Medium	Easy	Easy	Medium

**Table 3:** List of compounds toxicity endpoints includes acute toxicity, hepatotoxicity, cytotoxicity, carcinogenicity, and mutagenicity of selected three compounds.

Classification	Target	Amb23604132	Amb23604659	Amb1153724
Oral toxicity	LD_50_ (mg/kg)	5000	5000	5000
	Toxicity Class	5	5	5
Organ toxicity	Hepatotoxicity	Inactive	Inactive	Inactive
Toxicity endpoints	Carcinogenicity	Inactive	Inactive	Inactive
	Mutagenicity	Inactive	Inactive	Inactive
	Cytotoxicity	Inactive	Inactive	Inactive

**Table 4:** List of the interaction between the selected three compounds and MERS-CoV S1-NTD protein found during the complex structure analysis and generated through the docking simulation.

Compound	Residues	Bond Distance (Å)	Category	Bond Types
Amb1153724	GLN37	2.93083	Hydrogen Bond	Conventional H-B
	TRP44	1.96777	Hydrogen Bond	Conventional H-B
	HIS81	2.39932	Hydrogen Bond	Conventional H-B
	LYS42	1.99029	Hydrogen Bond	Conventional H-B
	GLN37	3.53458	Hydrogen Bond	Carbon H-B
	ASN104	3.56047	Hydrogen Bond	Carbon H-B
	ASP41	4.46727	Electrostatic	Pi-Anion
	MET84	2.6909	Hydrogen Bond	Pi-Donor H-B
	TYR314	5.61379	Hydrophobic	Pi-Pi T-shaped
	MET84	4.9701	Hydrophobic	Pi-Alkyl
	MET84	4.95866	Hydrophobic	Pi-Alkyl
Amb23604132	GLN37	2.54483	Hydrogen Bond	Conventional H-B
	LYS42	2.04393	Hydrogen Bond	Conventional H-B
	MET84	2.12397	Hydrogen Bond	Conventional H-B
	PHE40	2.49011	Hydrogen Bond	Conventional H-B
	ASP108	3.4188	Hydrogen Bond	Carbon H-B
	ASP108	4.6773	Electrostatic	Pi-Anion
	TYR314	5.74845	Hydrophobic	Pi-Pi T-shaped
	MET161	4.84458	Hydrophobic	Alkyl
	MET84	4.15196	Hydrophobic	Pi-Alkyl
	MET84	5.10244	Hydrophobic	Pi-Alkyl
Amb23604659	GLN37	2.99559	Hydrogen Bond	Conventional H-B
	ARG46	2.60436	Hydrogen Bond	Conventional H-B
	MET84	2.18527	Hydrogen Bond	Conventional H-B
	ASP41	2.59464	Hydrogen Bond	Conventional H-B
	ASP108	4.05492	Electrostatic	Pi-Anion
	MET161	3.87544	Hydrophobic	Pi-Sigma
	MET84	4.80802	Hydrophobic	Pi-Alkyl
	MET84	5.05152	Hydrophobic	Pi-Alkyl

## Data Availability

Not applicable.

## Supplementary Materials

The following are available online, Figure S1: Showing the blood brain barrier (BBB) and P-gp P-glycoprotein (P-GP) substrate activity of the selected four compounds, Amb6600135, Amb1153724, Amb23604132, and Amb23604659; Figure S2: Showing the contact mapping of the protein-ligands interactions for the selected three compounds found during the 200 ns simulation run. Herein, showing the selected three ligands (A) Amb23604659, (B) Amb23604132, and (C) Amb1153724 contact map with the desire S1-NTD protein; Figure S3: Depicted the RMSD ((Å), rGyr (Å), intra-HB, MolSA (Å2), SASA (Å2), and PSA (Å2 of the selected three compounds in complex with the MERS-CoV S1-NTD protein. Herein, showing the value of the compounds (A) Amb23604659, (B) Amb23604132, and (C) Amb1153724; Figure S4: The RMSD values the selected three complex structures and Folic acid in complex with the protein S1-NTD (PDB:5VYH). Herein, showing the RMSD of the compounds Amb23604659 (orange), Amb23604132 (Yellow), Amb1153724 (gray), and folic acid (blue) colors; and Figure S5: Depicted the torsion properties of the selected three compounds (A) Amb23604659, (B) Amb23604132, and (C) Amb1153724 during the 200 ns MD simulation run, Table S1: A list of 32 compounds generated as hits during pharmacophore based virtual screening process with there geometric fit score, conformation number and binding affinity (kcal/mol) with desire protein; Table S2: List of HOMO, LUMO, HOMO-LUMO gap, softness and hardness of the selected three compound; and Table S3: List of MM/GBSA component and their energy with standard error value of the selected three compound.
