# Supplementary material for: Pharmacophore-Based Virtual Screening, Quantum Mechanics Calculations, and Molecular Dynamics Simulation Approaches Identified Potential Natural Antiviral Drug Candidates against MERS-CoV S1-NTD
Source: Molecules. 2021 Aug 17;26(16):4961. doi: 10.3390/molecules26164961 (PMC8401589; doi:10.3390/molecules26164961)
Supplement: Supplementary file 1 [file molecules-26-04961-s001.zip › Supplementary Table.pdf]

Supplementary Table on

Pharmacophore-based virtual screening, quantum mechanics calculations, and molecular dynamics simulation approaches identified potential natural antiviral drug candidates against MERS-CoV S1-NTD.

Thamer Ahmad Bouback [1], Abdulaziz Albeshri [1], Amal Mohammed Aljohani [1], Abdus Samad [2, 3], Rahat Alam [2, 3], Md Saddam Hossen [2,3,4], Khalid Al-Ghamdi [1], Foysal Ahammad [1, 2, 3, *], Ishtiaq Qadri [1, **]

Table S1: A list of 32 compounds generated as hits during pharmacophore based virtual screening process with there geometric fit score, conformation number and binding affinity (kcal/mol) with desire protein.

Compound Name	Pharmacophore Fit Score	Number of conformations	Binding Affinity
**Amb6600135**	66.6	25	-9.2
**Amb23604132**	65.48	25	-9.1
**Amb23604659**	65.55	25	-8.6
**Amb1153724**	66.64	25	-8.1
**Amb24326239**	67.57	25	-8.0
**Amb22172700**	67.12	12	-7.7
**Amb22407028**	67.12	8	-7.7
**Amb19746939**	67.51	25	-7.6
**Amb24326238**	66.88	25	-7.6
**Amb28533232**	65.46	25	-7.6
**Amb21855756**	66.6	25	-7.5
**Amb22407027**	67.12	14	-7.5
**Amb22735040**	66.88	25	-7.5
**Amb22800823**	66.21	25	-7.5
**Amb29085648**	66	16	-7.5
**Amb22888458**	66.84	25	-7.4
**Amb29085659**	67.1	25	-7.4
**Amb29085660**	66.85	25	-7.4
**Amb22584586**	66.83	25	-7.3
**Amb18003253**	66.77	25	-7.2
**Amb29085723**	66.83	25	-7.2
**Amb19886350**	67.46	25	-7.1
**Amb19893465**	66.38	25	-7.1
**Amb24323825**	66.81	25	-7.1
**Amb28532929**	66.59	25	-7.1
**Amb29085710**	67.75	25	-7.1
**Amb19928430**	65.71	9	-6.9
**Amb22584350**	66.54	25	-6.9
**Amb22888476**	66.95	25	-6.9
**Amb22896309**	66.27	25	-6.7
**Amb8514723**	66.58	25	-6.6
**Amb22584735**	66.63	25	-6.4

Table S2: List of HOMO, LUMO, HOMO-LUMO gap, softness and hardness of the selected three compound.

Compound Name	HOMO (a.u)	LUMO (a.u)	Area (HOMO)	Area (LUMO)	HLG (a.u)	Softness	Hardness	Iso-value
Amb1153724	-0.207	-0.0425	70.947	62.03	0.1645	0.0822	12.157	-0.05
Amb23604132	-0.211	-0.079	107.067	85.507	0.1326	0.0663	15.084	-0.05
Amb23604659	-0.217	-0.057	120.442	58.779	0.1601	0.0801	12.488	-0.05

Table S3: List of MM/GBSA component and their energy with standard error value of the selected three compound.

Compound Name	ΔG Bind	ΔG Bind Coulomb	ΔG Bind Covalent	ΔG Bind Hbond	ΔG Bind Lipo	ΔG Bind Packing	ΔG Bind Solv GB	ΔG Bind vdW
**Amb1153724**	-32.47±6	-22.45±4	2.5±1	-2.08±1	-7.51±3	-6.91±2	16.2±4	-14.22±4
**Amb23604132**	-24.75±4	-12.25±5	1.41±.5	-1.01±.6	-7.28±2	-9.79±3	16.28±5	-10.3±3
**Amb23604659**	-26.18±5	-6.43±4	1.7±.6	-1.04±.5	-5.4±3	-5.86±1	10.03±3	-17.78±7
