# Supplementary material for: Pharmacophore-Based Virtual Screening, Quantum Mechanics Calculations, and Molecular Dynamics Simulation Approaches Identified Potential Natural Antiviral Drug Candidates against MERS-CoV S1-NTD
Source: Molecules. 2021 Aug 17;26(16):4961. doi: 10.3390/molecules26164961 (PMC8401589; doi:10.3390/molecules26164961)
Supplement: Supplementary file 1 [file molecules-26-04961-s001.zip › Supplementary Figure.pdf]

Supplementary figure on

Pharmacophore-based virtual screening, quantum mechanics calculations, and molecular dynamics simulation approaches identified potential natural antiviral drug candidates against MERS-CoV S1-NTD.

Thamer Ahmad Bouback [1], Abdulaziz Albeshri [1], Amal Mohammed Aljohani [1], Abdus Samad [2, 3], Rahat Alam [2, 3], Md Saddam Hossen [2,3,4], Khalid Al-Ghamdi [1], Foysal Ahammad [1, 2, 3, *], Ishtiaq Qadri [1, **]

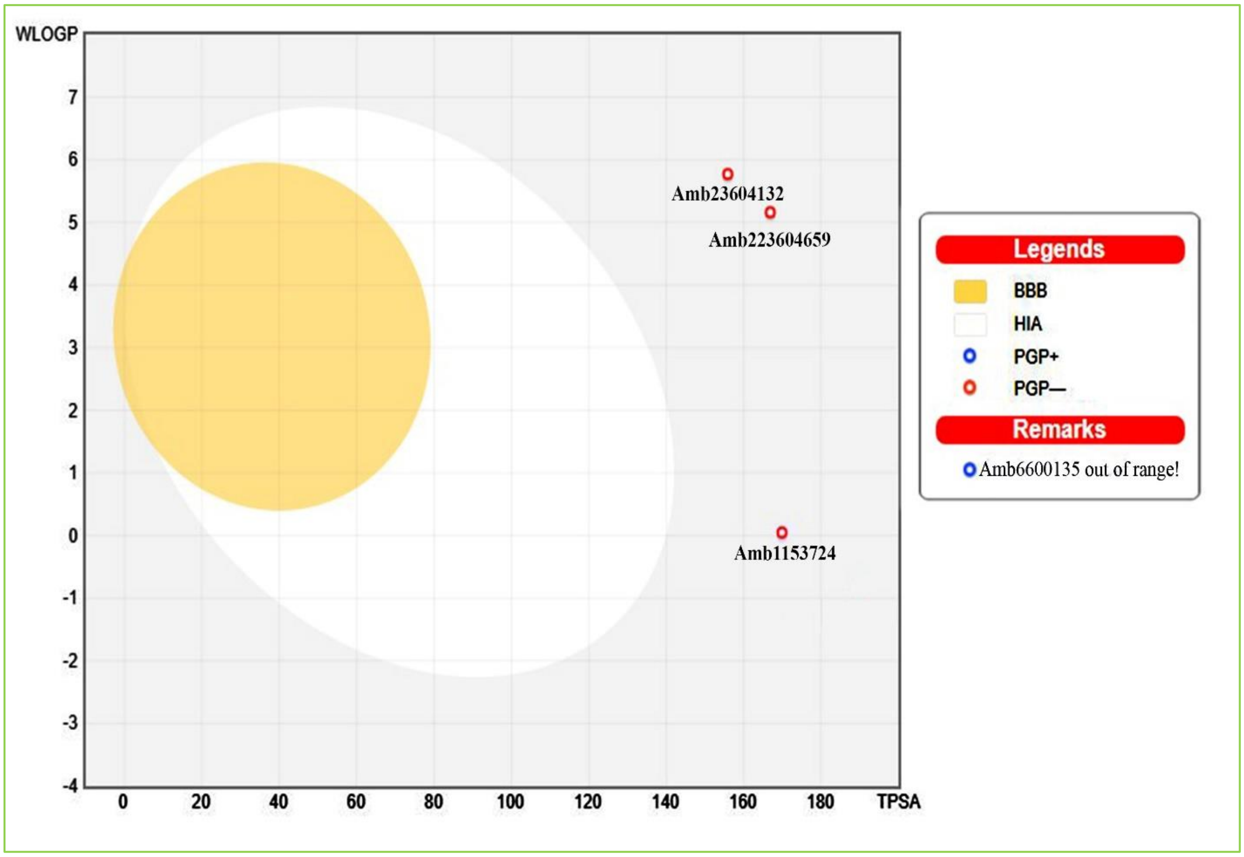

Figure S1: Showing the blood brain barrier (BBB) and P-gp P-glycoprotein (P-GP) substrate activity of the selected four compounds, Amb6600135, Amb1153724, Amb23604132, and Amb23604659.

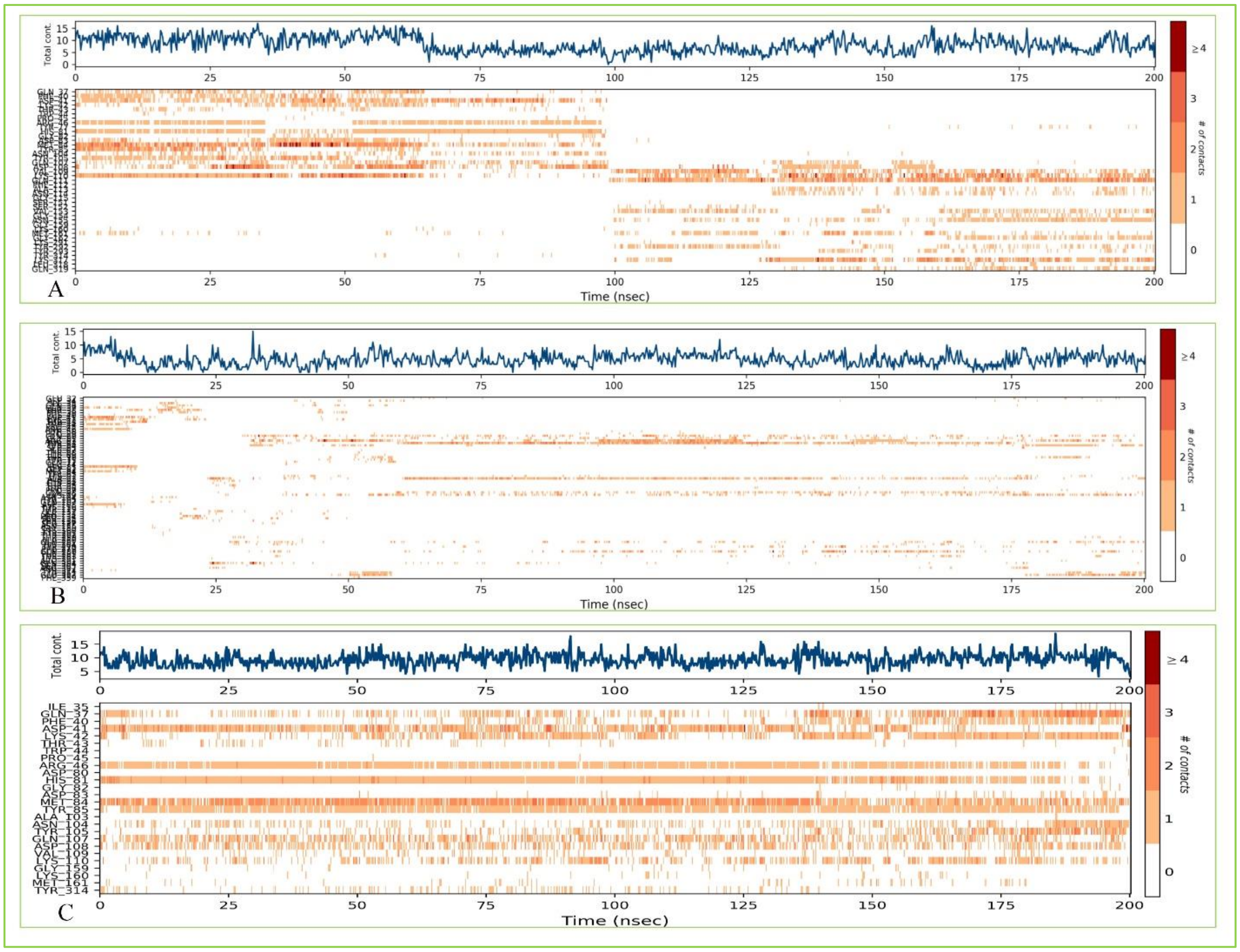

Figure S2: Showing the contact mapping of the protein-ligands interactions for the selected three compounds found during the 200 ns simulation run. Herein, showing the selected three ligands (A) Amb23604659, (B) Amb23604132, and (C) Amb1153724 contact map with the desire S1-NTD protein.

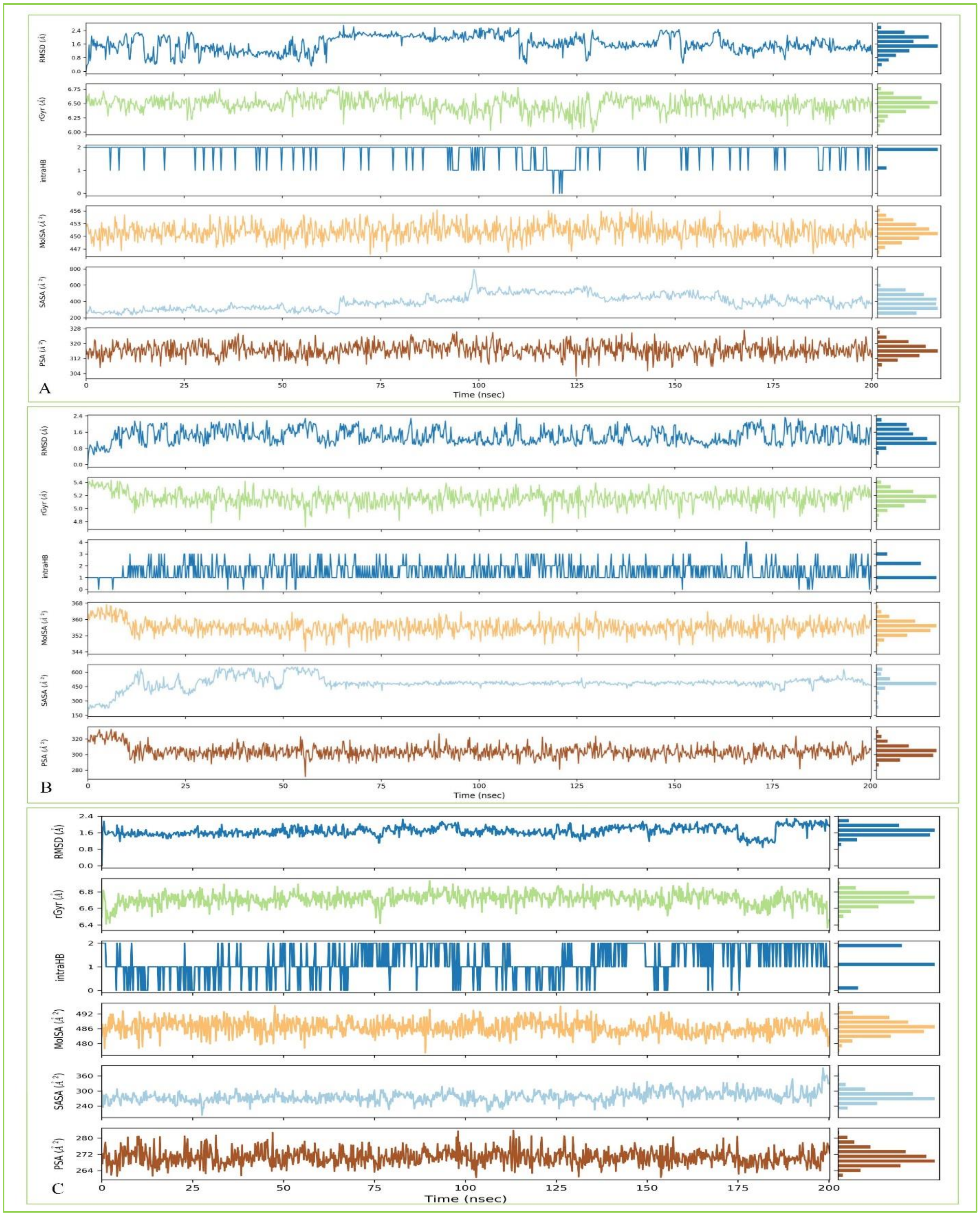

Figure S3: Depicted the RMSD ((Å), rGyr (Å), intra-HB, MolSA(Å^2), SASA(Å^2), and PSA (Å^2 of the selected three compounds in complex with the MERS-CoV S1-NTD protein. Herein, showing the value of the compounds (A) Amb23604659, (B) Amb23604132, and (C) Amb1153724.

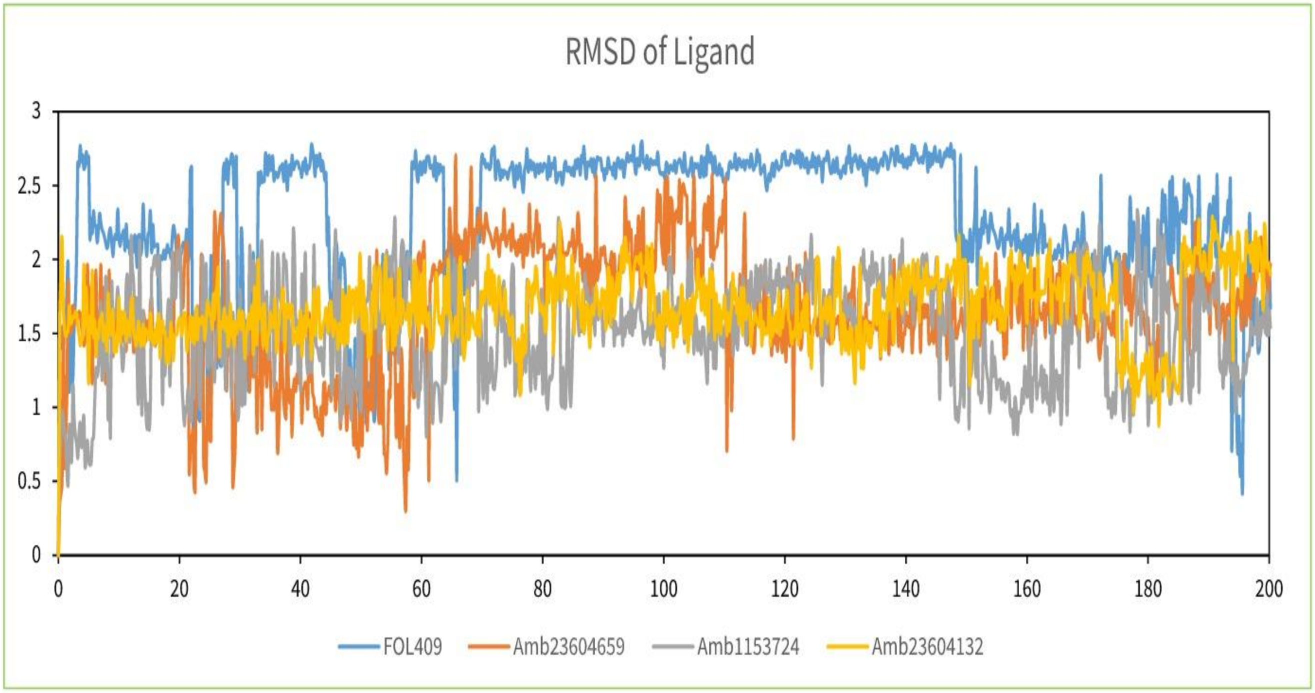

Figure S4: The RMSD values the selected three complex structures and Folic acid in complex with the protein S1-NTD (PDB:5VYH). Herein, showing the RMSD of the compounds Amb23604659 (orange), Amb23604132 (Yellow), Amb1153724 (gray), and folic acid (blue) colors.

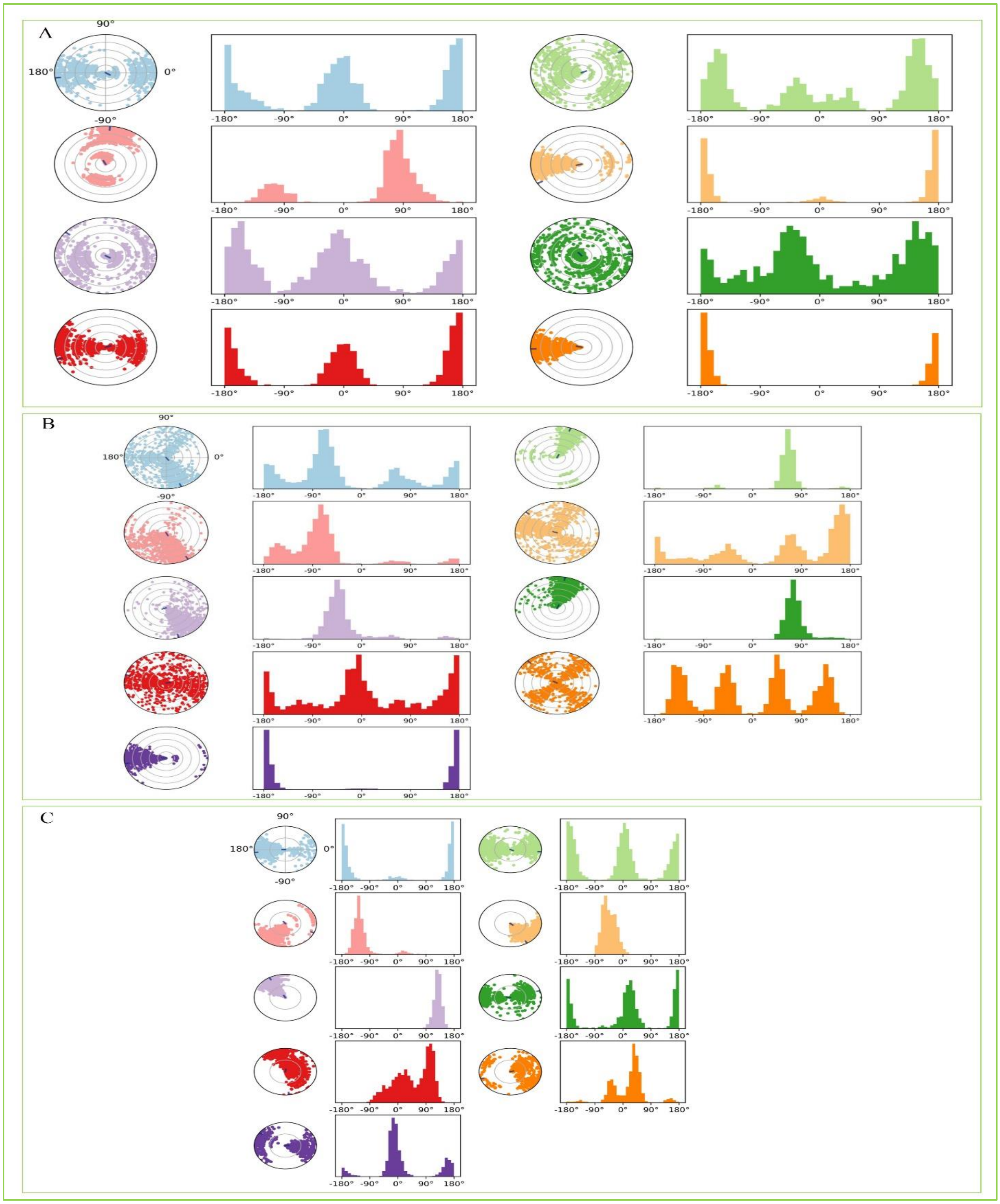

Figure S5: Depicted the torsion properties of the selected three compounds (A) Amb23604659, (B) Amb23604132, and (C) Amb1153724 during the 200 ns MD simulation run.
