# Supplementary material for: Pharmacophore-Based Virtual Screening, Quantum Mechanics Calculations, and Molecular Dynamics Simulation Approaches Identified Potential Natural Antiviral Drug Candidates against MERS-CoV S1-NTD
Source: Molecules. 2021 Aug 17;26(16):4961. doi: 10.3390/molecules26164961 (PMC8401589; doi:10.3390/molecules26164961)
Supplement: Supplementary file 1 [file molecules-26-04961-s001.zip › English Editing-Certificate.pdf]

CERTIFICATE OF ENGLISH EDITING

This certificate confirms that the document listed below was edited by a professional editor working on behalf of FirstEditing.com. The editing project was performed at:

Level 3 Premium Content Editing

This editing level includes the following services:

Level 3 - Premium Content Editing: Content Editing includes Editing Levels 1 and 2 (spelling, grammar, punctuation, consistency/appropriateness of verb tenses, transitional phrasing, continuity/flow of thoughts, support of statements, formatting of citations/references, and footnote editing) PLUS supplementary sentence re-writes when necessary.

Document Title:

“Pharmacophore-based virtual screening, quantum mechanics calculations, and molecular dynamics simulation approaches identified potential natural antiviral drug candidates against MERS-CoV S1-NTD”

Date Edited:

April 21, 2021

FirstEditing.com specializes in the comprehensive editing of scientific and academic documents, targeted for publication in English language journals. FirstEditing.com provides editing and formatting services for theses and dissertations, scientific and academic documents, essays, research papers, grant proposals, and other scientific materials. Every order is edited by one or more professional academic editor with an advanced academic degree. All editors are native English speakers.
